# Direct healthcare costs of non-metastatic castration-resistant prostate cancer in Italy

**DOI:** 10.1017/S0266462322003336

**Published:** 2023-01-06

**Authors:** Ludovica Borsoi, Oriana Ciani, Giuseppe Fornarini, Marco Oderda, Alessandro Sciarra, Damir Vetrini, Irene Luccarini

**Affiliations:** 1 Centre for Research on Health and Social Care Management (CERGAS), SDA Bocconi School of Management, Milan, Italy; 2 Medical Oncology Unit 1, IRCCS Ospedale Policlinico San Martino, Genoa, Italy; 3 Department of Urology, Città della Salute e della Scienza di Torino – Molinette Hospital, University of Turin, Turin, Italy; 4 Prostate Cancer Unit, Department of Urology, Policlinico Umberto I, Sapienza University, Rome, Italy; 5 Janssen-Cilag S.p.A., Cologno Monzese, Italy

**Keywords:** cost of illness, expert elicitation, castration-resistant prostate, cancer, mixed-methods, direct healthcare costs

## Abstract

**Objectives:**

The management of non-metastatic castration-resistant prostate cancer (nmCRPC) is rapidly evolving; however, little is known about the direct healthcare costs of nmCRPC. We aimed to estimate the cost-of-illness (COI) of nmCRPC from the Italian National Health Service perspective.

**Methods:**

Structured, individual qualitative interviews were carried out with clinical experts to identify what healthcare resources are consumed in clinical practice. To collect quantitative estimates of healthcare resource consumption, a structured expert elicitation was performed with clinical experts using a modified version of a previously validated interactive Excel-based tool, EXPLICIT (EXPert eLICItation Tool). For each parameter, experts were asked to provide the lowest, highest, and most likely value. Deterministic and probabilistic sensitivity analyses (PSA) were carried out to test the robustness of the results.

**Results:**

Ten clinical experts were interviewed, and six of them participated in the expert elicitation exercise. According to the most likely estimate, the yearly cost per nmCRPC patient is €4,710 (range, €2,243 to €8,243). Diagnostic imaging (i.e., number/type of PET scans performed) had the highest impact on cost. The PSA showed a 50 percent chance for the yearly cost per nmCRPC patient to be within €5,048 using a triangular distribution for parameters, and similar results were found using a beta-PERT distribution.

**Conclusions:**

This study estimated the direct healthcare costs of nmCRPC in Italy based on a mixed-methods approach. Delaying metastases may be a reasonable goal also from an economic standpoint. These findings can inform decision-making about treatments at the juncture between non-metastatic and metastatic prostate cancer disease.

## Introduction

Cost-of-illness (COI) studies are intended to assess the costs associated with a specified illness and perspective (1). They provide estimates of the costs associated with treating and managing illnesses paid for by patients, governments, insurers, and charitable organizations. Using primary data collection, through interviews or surveys, they reflect the economic burden of disease and uncover gaps in the health system, by pointing out diseases that consume the most resources and possibly opportunities for saving (2). In this study, we rely on this methodological tool to investigate a previously overlooked patient population, which is non-metastatic castration-resistant prostate cancer (nmCRPC), with a focus on costs borne by the National Healthcare System (NHS).

Prostate cancer (PCa) is the second most commonly diagnosed cancer and the fifth most common cause of cancer death in men worldwide, accounting for 1,276,106 incident cases and 358,989 deaths in 2018 (3). Although the incidence of PCa has more than tripled between 1990 and 2015 (4), also due to an increase in the diffusion of prostate-specific antigen (PSA) screening that aided in early cancer detection and diagnosis (5), mortality rates have been decreasing in most Western countries thanks to improved diagnosis and treatment (6). In Italy, PCa is the most commonly diagnosed cancer in men (19 percent of cancer diagnoses) and the third most common cause of cancer death (8 percent of deaths), with a 5 and 10-year survival probability of 92 percent and 90 percent, respectively (7).

Treatment options for PCa are frequently updated and depend on prognostic factors, including age, stage of disease, comorbidities, and life expectancy. Often PCa is diagnosed as a localized disease and its management includes curative intent therapies such as surgery (radical prostatectomy), radiotherapy, chemotherapy (as neoadjuvant therapy in localized high-risk PCa), or combination approaches such hormonal therapy (androgen-deprivation therapy, ADT) before prostatectomy (8). Although PCa patients undergoing first-line treatments have high cure rates, some of them will experience progression, either through biochemical recurrence or progression to metastatic disease (9;10). In patients suffering from disease relapse, delaying metastases development is a key therapeutic goal as it may decrease cancer-related complications and enhance survival (11;12). Castration through androgen deprivation therapy (ADT), by blocking the production of testosterone, leads to decreased proliferation of cancer cells and should prevent disease progression (13). Although systemic hormone therapy is initially effective in reducing disease progression, patients eventually develop a castration-resistant state (CRPC), characterized by rising PSA during active treatment (14). If PSA progression during treatment with ADT occurs prior to the detection of metastases, patients enter an nmCRPC state (15), defined by serum testosterone <50 ng/dL and rising PSA in the absence of radiographic evidence of metastatic disease (16). Recently, three next-generation anti-androgens (i.e., enzalutamide, apalutamide, and darolutamide) that target the androgen receptor were tested in international, randomized, placebo-controlled, phase III trials (17–19) and approved by the Food and Drug Administration (FDA) and the European Medicines Agency (EMA). All these drugs significantly increased metastasis-free survival (MFS), a surrogate outcome for overall survival in clinical studies of men with localized PCa (11;20), and overall survival (21–23).

Effective management of nmCRPC is essential in order to delay metastases development, as it substantially increases both clinical burden and costs associated with the disease (24). However, diagnostic and treatment pathways and monitoring activities for these patients may vary substantially in clinical practice at the national level. Moreover, despite growing concern about nmCRPC from a public health perspective, its direct healthcare costs have never been estimated in Italy, thus limiting the possibility for decision-makers of assessing the beneficial impact of new therapies in reducing the costs associated with the development of metastases. This study aims at filling this gap by estimating the COI of nmCRPC treated with standard ADT from the Italian NHS perspective, with the ultimate aim of informing evidence-based decision-making and healthcare prioritization policies that will guarantee an appropriate resource use for this patient population.

## Methods

The study was conducted through two key methodological steps: a qualitative interview-based component and a quantitative expert elicitation component. Ethical approval for this research was obtained from the Ethics Committee of Università Commerciale Luigi Bocconi in September 2019.

## Interviews

Structured, individual qualitative interviews were carried out with a selected sample of clinical experts, medical oncologists, and urologists, aimed at identifying the types of healthcare resources consumed (e.g., outpatient visits, laboratory tests, ADT treatment, management of adverse events) by nmCRPC patients in the current clinical practice in order to inform the COI analysis. The interview guide is provided in the Supplementary File 1. Clinicians were selected through a purposive sampling on the basis of their recognized expertise in the management of nmCRPC (e.g., authors of peer-reviewed publication on nmCRPC) and in order to reflect current practice and geographical representativeness across different Italian regions. A pilot phase of interviews was scheduled with two experts in order to ensure validity and clear wording of the questions. Interviews were conducted in Italian either by phone or face-to-face according to clinicians’ preferences and availability. Interviews were carried out between October and November 2019 (i.e., before the Italian Medicines Agency’s reimbursement decisions for next-generation antiandrogens). Consent was gained from all interviewees to partake in the project.

## Expert elicitation and COI analysis

To assess the direct healthcare costs of nmCRPC treated with standard ADT in Italy from the perspective of the NHS and with a 1-year time-horizon, we carried out a structured expert elicitation exercise and collected several quantitative estimates of healthcare resource consumption. We chose 1-year time horizon for two main reasons. The first is a more methodological one, as whilst asking clinicians to provide their opinion, we thought that limiting their estimates of resource consumption over a clearly predefined (1-year) time horizon facilitated their answers and the inter-expert comparability. The second is more practical, as this is the time horizon also used by other studies which estimated healthcare costs of nmCRPC (always reporting the per-patient-per-year cost) (25–27).

An initial list of relevant healthcare resources was drafted based on the clinical literature (17;18). For example, AEs with grade ≥ 3 due to ADT sourced from two international RCTs (17;18) were investigated during the interviews. The list was further refined on the basis of information collected during qualitative interviews. A review of epidemiological data around nmCRPC in Italy was conducted revealing scant available evidence that may be explained by the difficulty in identifying the target population from a clinical point of view. A recent model-based study provided prevalence data of PCa at different clinical stages in Italy; however, authors did not report separate estimates for the nmCRPC population (10).

Expert elicitation is the process of obtaining probabilistic belief statements from experts about unknown quantities or parameters, and it represents a useful approach when data from empirical evidence or published literature are either scant or unavailable (28). Clinical experts involved in the qualitative interviews were invited to participate in the expert elicitation exercise. As regards the elicitation method, we used a 3-point estimation, that is, we elicited the lowest (L), highest (H), and most likely value (M) of the quantity of interest. Other studies published on expert elicitation use the “complementary intervals” or hybrid method (29;30). In this method, intervals (usually 4) are created between the three elicited values (i.e., L, H, and M) and experts are asked to specify the probability that their estimated value lies within each interval. However, as we elicited experts’ opinion on a relevant number of parameters (approximately 30), we asked clinicians to provide only the three estimates, that is L, H, and M values, in order to decrease the burden of compilation. To carry out the expert elicitation exercise, we used a modified version of a previously validated Excel-based tool, EXPLICIT (EXPert eLICItation Tool), developed by researchers of the University of Exeter Medical School (31). EXPLICIT is a self-administered elicitation tool that experts could use by themselves either with some assistance (in face-to-face elicitation) or no assistance (in distance via mail elicitation). The original EXPLICIT tool (available from authors (31)) was modified and adapted to the objectives of the present study and an Italian version was created. The tool was composed by three main sections: (i) an introduction section, providing summary information on the study objectives and methods, as well as an electronic consent form that experts must fill in in order to participate in the exercise; (ii) a training section, that prepares the expert for the elicitation task by providing instructions on how to give estimates and provides an example question; (iii) the questionnaire section. In the latter section, experts were asked to provide – for each parameter of interest – the L, H, and M values. Parameters were classified according to the following sub-sections: (i) diagnosis; (ii) follow-up; (iii) ADT treatment; (iv) management of AEs. Clinicians were asked to indicate either the number of resources consumed in 1 year by an nmCRPC patients (e.g., number of outpatient visits) or the percentage of patients consuming a certain resource (e.g., percent of patients treated with a certain ADT).

Once the values were provided, the tool verified their completeness and consistency through a series of macros (e.g., that the L estimate was lower than the H one) and represented them through a graph (i.e., the probability density function of a triangular distribution obtained from the three estimates), in order to ensure that the experts were satisfied with the graphical representation of their estimate. The tool was constructed in November 2019, and a pilot phase was scheduled in early December 2019 with two health economists, experts in economic evaluation analysis, in order to test the new version of the tool. In the pilot phase, health economists were asked to fill in the tool in order to verify whether: (i) questions were comprehensible and unambiguous; (ii) graphs were correctly created after estimates provision; (iii) macros were properly working (e.g., they were able to identify inconsistencies in estimates and incomplete fields); (iv) the Excel file could be opened on different devices; (v) the completion time was appropriate. After the pilot, minor adjustments (e.g., wording, correction of a macro) were made to the tool. Sample screens of the tool (in Italian) are shown in Supplementary File 2. The tool was then sent by email to the experts involved, who were supported by phone in case of need in order to facilitate exercise completion. Data were analyzed by averaging the estimates provided by experts, that is, we aggregated separately the three values L, H, and M. All experts were given equal weight in data aggregation. We believed that weighting experts equally was more prudent than weighting them by the precision of their elicited values as we do not know whether that precision was an accurate reflection of an expert’s actual knowledge about a certain parameter (32).

Alongside the collection of healthcare resource consumption estimates from experts, unit costs of resources were retrieved to carry out the COI analysis. The unit costs of diagnostic tests, outpatient visits and laboratory tests were sourced from “Nomenclatore dell’assistenza specialistica ambulatoriale”, an official document published on the Italian Ministry of Health website providing national tariffs (updated to 2013) for outpatient services (33). As regards treatment costs, we derived the official ex-manufactory price per mg (including the mandatory 5 percent+5 percent discount) from a database provided by Farmadati, which collects and constantly updates all information related to official drug prices in Italy. Finally, the management of severe adverse events was valued through Italian DRG tariffs (34).

The yearly cost of an nmCRPC patient for the Italian NHS was calculated by multiplying the resource consumption estimates provided by experts by their respective unit cost. As we elicited different estimates from experts, namely the L, H, and M value, we provided three different scenarios: (i) the base-case scenario, in which M values were considered; (ii) the most conservative scenario, in which L values were considered; (iii) the least conservative scenario, in which H values were considered.

Sensitivity analyses were performed to assess how parameters’ uncertainty affected the results. Deterministic sensitivity analysis (DSA) was carried out using the base-case scenario as a reference, and varying each parameter elicited from experts according to its minimum (L) and maximum (H) estimate. Results were graphically represented through a tornado diagram. Probabilistic sensitivity analysis (PSA) was performed on all elicited parameters using a Monte Carlo simulation with 1,000 iterations. Parameters were varied according to first to a triangular distribution, then to a beta-PERT distribution. Results were reported through a histogram for both distributions used, which provided information on the frequency distribution of total cost estimates.

## Results

**Figure 1.**
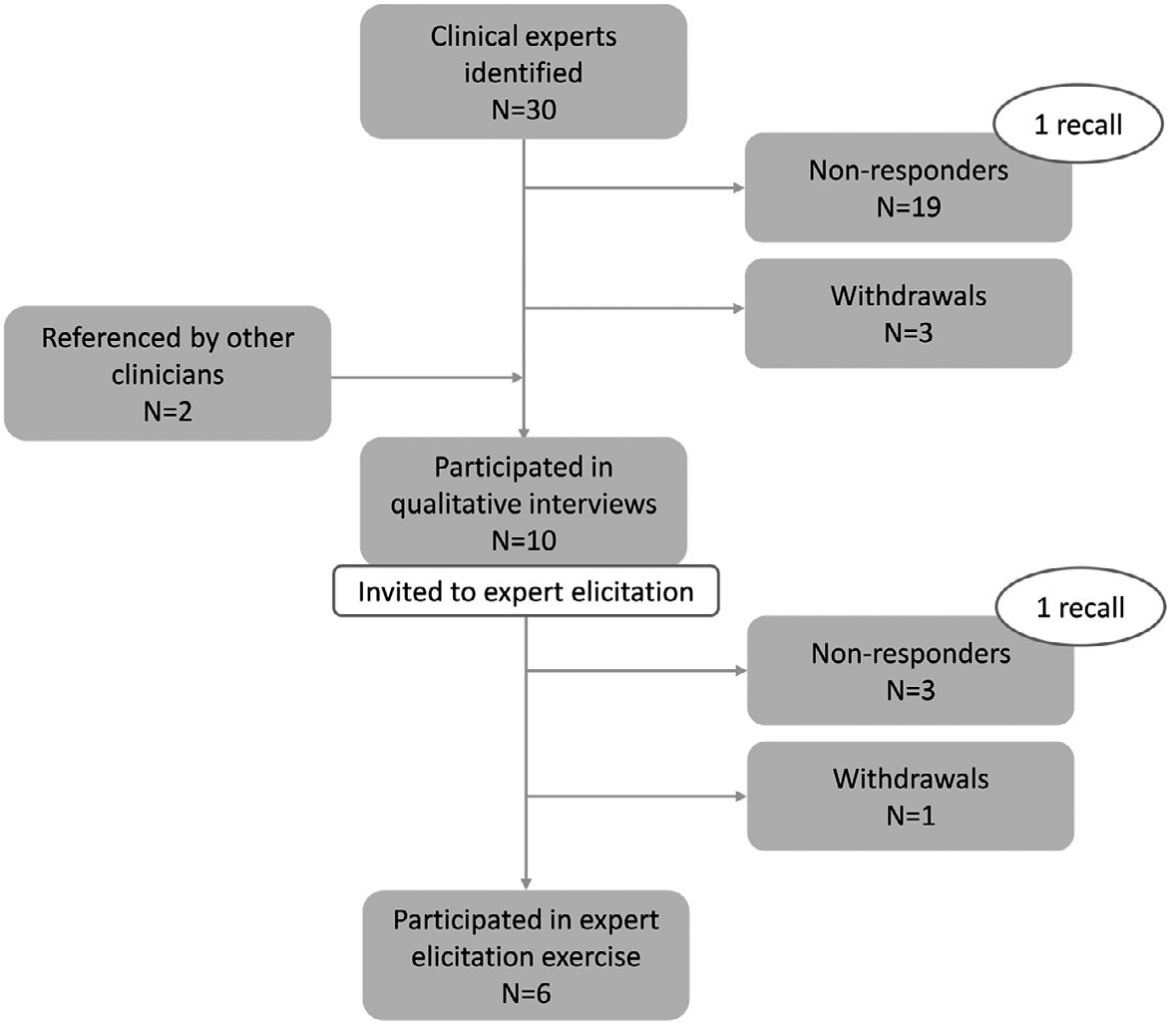
Sample identification process for qualitative interviews and expert elicitation.

Thirty clinical experts were identified based on their scientific publications and their recognized experience in the treatment of nmCRPC and were invited via email to participate in the research. Two experts were referenced by contacted clinicians and invited. Ten experts (31 percent), four urologists, and six oncologists operating in eight different Italian regions with an average experience of 13 years in the management of patients with PCa agreed to be interviewed (Figure 1). Interviews revealed that the management of nmCRPC is heterogeneous across Italian regions and hospitals. According to the interviewees, healthcare resource consumption of nmCRPC patients is mostly limited to imaging, follow-up visits in an outpatient regimen with their clinician (urologist, oncologist or radiotherapist), laboratory tests (e.g., PSA, testosterone, blood count), and hormonal therapy. Overall, clinicians reported few serious AEs experienced by their nmCRPC patients treated with ADT alone. Moreover, most of the AEs were experienced by a neglectable proportion of the population and did not entail the consumption of costly resources (e.g., diarrhea, constipation, and dizziness).

Among the clinicians interviewed, six, four urologists and two oncologists, participated in the expert elicitation exercise (response rate = 60 percent) (Figure 1). Among the clinicians who did not adhere to this project phase, two declared that they could not participate due to lack of time, and two of them never replied to the invitation email. All clinicians who confirmed their willingness to participate completed the elicitation exercise (completion rate = 100 percent). The tool completion required approximately 20 minutes. Although the majority of clinicians did not provide any feedback after their participation in the expert elicitation, some of them declared that it was an interesting experience, as they never participated to such an exercise. Five clinicians out of six completed the tool autonomously, and they did not report any difficulty in carrying out the exercise. One clinician, due to technical issue with his computer, was supported in the completion by one of the authors via Microsoft Teams.

Table 1 reports the list of healthcare resources consumed by nmCRPC patients, drafted based on individual interviews. Moreover, for each resource, it reports the mean and standard deviation of consumption estimates (L, H, and M value) provided by experts related to diagnosis, follow-up, treatment, and AEs management of nmCRPC patients treated with ADT in the Italian NHS. Overall, the estimates provided were quite similar across respondents for the majority of parameters. For some of the parameters (e.g., number of outpatient visits, percent of patients hospitalized for urinary retention and major cardiovascular events, some laboratory tests), we noticed a higher heterogeneity in estimates, with the presence of some outliers. However, it is worth underlining that these outliers were distributed across clinicians’ estimates, that is, none of the clinicians repeatedly provided extreme values.

**Table 1.** Expert estimates for healthcare resource consumption

Parameter	Most likely (M) estimate	Lowest (L) estimate	Highest (H) estimate
	mean (SD)	mean (SD)	mean (SD)
*Diagnosis*			
# CT scan/year	1.17 (0.75)	0.83 (0.75)	2.67 (1.03)
# bone scan/year	1.00 (0.63)	0.50 (0.55)	1.83 (0.75)
# PET choline/year	1.08 (0.80)	0.50 (0.55)	1.83 (0.75)
# PET PSMA/year	1.08 (0.80)	0.50 (0.55)	1.83 (0.75)
*Follow-up (visits and laboratory tests)*			
# Outpatient visit/year	5.17 (3.43)	3.83 (2.23)	7.67 (3.61)
# PSA test/year	5.00 (1.67)	3.50 (1.52)	8.67 (3.01)
# Testosterone test/year	3.83 (2.64)	2.50 (2.17)	6.33 (3.44)
# Blood count test/year	3.83 (4.02)	2.00 (1.10)	5.33 (4.46)
# Blood glucose test/year	3.33 (4.27)	1.67 (1.21)	4.50 (4.72)
# Calcium test/year	1.83 (1.17)	1.17 (0.98)	3.17 (2.04)
# Vitamin D test/year	1.83 (0.98)	1.00 (0.89)	3.17 (1.72)
# Lipid profile test/year	2.00 (2.28)	0.83 (0.98)	4.00 (4.15)
# Creatinine test/year	2.67 (1.63)	1.50 (0.55)	5.00 (3.69)
# Electrolyte test/year	2.17 (0.98)	1.50 (0.55)	3.67 (1.37)
# Transaminases test/year	3.67 (4.32)	1.67 (1.03)	5.00 (4.65)
# Alkaline phosphatase test/year	1.67 (1.63)	1.17 (0.98)	2.67 (2.16)
# Bilirubin test/year	3.00 (4.47)	1.33 (0.82)	4.17 (4.88)
*ADT treatment*			
% Treated with buserelin	1.67 (4.08)	0.00 (0.00)	10.17 (19.90)
% Treated with goserelin	1.67 (4.08)	0.00 (0.00)	10.17 (19.90)
% Treated with leuprorelin	50.00 (18.17)	28.33 (17.22)	66.67 (24.22)
% Treated with triptorelin	44.17 (8.01)	25.00 (12.25)	65.00 (24.29)
% Treated with degarelix	14.83 (9.17)	9.50 (10.84)	28.83 (17.89)
% Yreated with bicalutamide	15.83 (11.14)	10.00 (8.94)	36.67 (24.22)
% Treated with flutamide	0.00 (0.00)	0.00 (0.00)	1.67 (4.08)
*Serious AEs*			
% Hospitalized for hematuria	0.42 (0.49)	0.17 (0.41)	2.67 (3.78)
% Hospitalized for urinary retention^a^	5.67 (11.98)	0.17 (0.41)	8.67 (15.82)
% Hospitalized for fractures	0.96 (1.10)	0.25 (0.42)	1.67 (1.97)
% Hospitalized for major cardiovascular events^a^	8.92 (15.34)	1.00 (0.89)	20.83 (25.53)

a The estimates provided by experts on the percentage of patients treated with ADT with severe urinary retention and major cardiovascular events are substantially higher than those reported in the trials from which these AEs were sourced. These real-world estimates may differ also due to more restricted sample of patients with nmCRPC, for whom the incidence of these AEs may appear higher.

ADT, androgen-deprivation therapy; AEs, adverse events; CT, computerized tomography; PET PSMA, prostate-specific membrane antigen ligand positron emission tomography; PSA, prostate-specific antigen.

Unit costs of healthcare resources related to diagnosis and follow-up are shown in Supplementary Table 1. On the basis of information provided by experts on the dosage and frequency of administration of ADT treatments (see Supplementary Table 2), the total therapy cost per year was calculated from the drug price per mg, as reported in Supplementary Table 3. As patients may be educated on self-administered drug injections or may receive the injection in a setting different from the hospital by other healthcare professionals (e.g., general practitioners, private nurses), we did not add any drug administration costs. To evaluate the management of serious AEs in monetary terms, we assumed that all patients were hospitalized for more than 1 day. In case more than one DRG could be assigned to a certain AE (see Supplementary Table 4), the average tariff was computed. Supplementary Table 5 shows the unit cost (i.e., average tariff) considered for the management of each AE.

Results provided in Table 2 show that the yearly cost per patient ranged from €2,243 to €8,243 (based on the L and H values elicited, respectively), with a most likely estimate equal to €4,710.

**Table 2.** Direct healthcare costs of non-metastatic castration-resistant prostate cancer (nmCRPC) in Italy (NHS perspective)

Parameter	Cost of nmCRPC per patient/year		
	Base-case scenario	Most conservative scenario	Least conservative scenario
*Diagnosis*			
CT scan	€ 92.72	€ 66.23	€ 211.92
Bone scan	€ 113.10	€ 56.55	€ 207.35
PET choline	€ 1,160.95	€ 535.83	€ 1,964.69
PET PSMA	€ 1,160.95	€ 535.83	€ 1,964.69
Total diagnosis costs	€ 2,527.72	€ 1,194.43	€ 4,348.65
*Follow-up (visits and laboratory tests)*			
Outpatient visit	€ 106.74	€ 79.20	€ 158.39
PSA test	€ 37.05	€ 25.94	€ 64.22
Testosterone test	€ 37.49	€ 24.45	€ 61.94
Blood count test	€ 12.15	€ 6.34	€ 16.91
Blood glucose test	€ 3.90	€ 1.95	€ 5.27
Calcium test	€ 2.07	€ 1.32	€ 3.58
Vitamin D test	€ 29.08	€ 15.86	€ 50.22
Lipid profile test	€ 4.42	€ 1.84	€ 8.84
Creatinine test	€ 3.01	€ 1.70	€ 5.65
Electrolyte test	€ 19.54	€ 13.53	€ 33.07
Transaminases test	€ 7.48	€ 3.40	€ 10.20
Alkaline phosphatase test	€ 1.73	€ 1.21	€ 2.77
Bilirubin test	€ 3.39	€ 1.51	€ 4.71
Total follow-up costs	€ 268.06	€ 178.24	€ 425.77
*ADT treatment*			
Buserelin	€ 21.44	€ 0.00	€ 130.76
Goserelin	€ 22.73	€ 0.00	€ 138.66
Leuprorelin	€ 557.60	€ 315.97	€ 743.47
Triptorelin	€ 533.46	€ 301.96	€ 785.10
Degarelix	€ 229.62	€ 147.06	€ 446.34
Bicalutamide	€ 84.70	€ 53.50	€ 196.16
Flutamide	€ 0.00	€ 0.00	€ 4.57
Total ADT treatment costs	€ 1,449.55	€ 818.49	€ 2,445.05
Serious AEs			
Hematuria	€ 6.15	€ 2.46	€ 39.37
Urinary retention	€ 83.67	€ 2.46	€ 127.96
Fractures	€ 34.97	€ 9.12	€ 60.82
Major cardiovascular events	€ 340.23	€ 38.16	€ 794.92
Total AEs management costs	€ 465.02	€ 52.20	€ 1,023.08
TOTAL COST	€ 4,710.36	€ 2,243.35	€ 8,242.55

*Note: M, L, and H values elicited from experts were used for base-case, most conservative and least conservative scenarios, respectively.*

ADT, androgen-deprivation therapy; AEs, adverse events; CT, computerized tomography; PET PSMA, prostate-specific membrane antigen ligand positron emission tomography; PSA, prostate-specific antigen.

**Figure 2.**
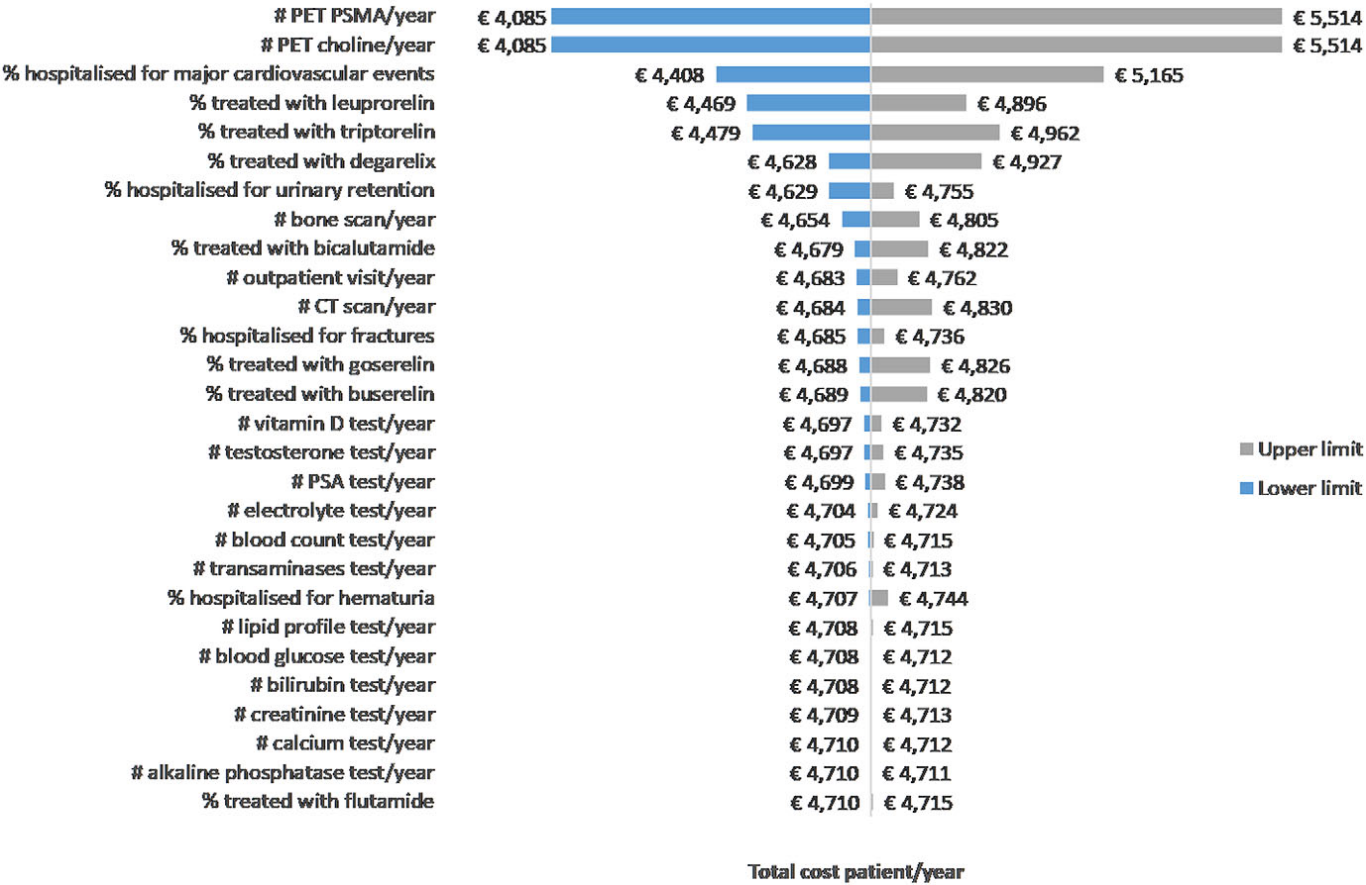
Deterministic sensitivity analysis – Tornado diagram.

**Figure 3.**
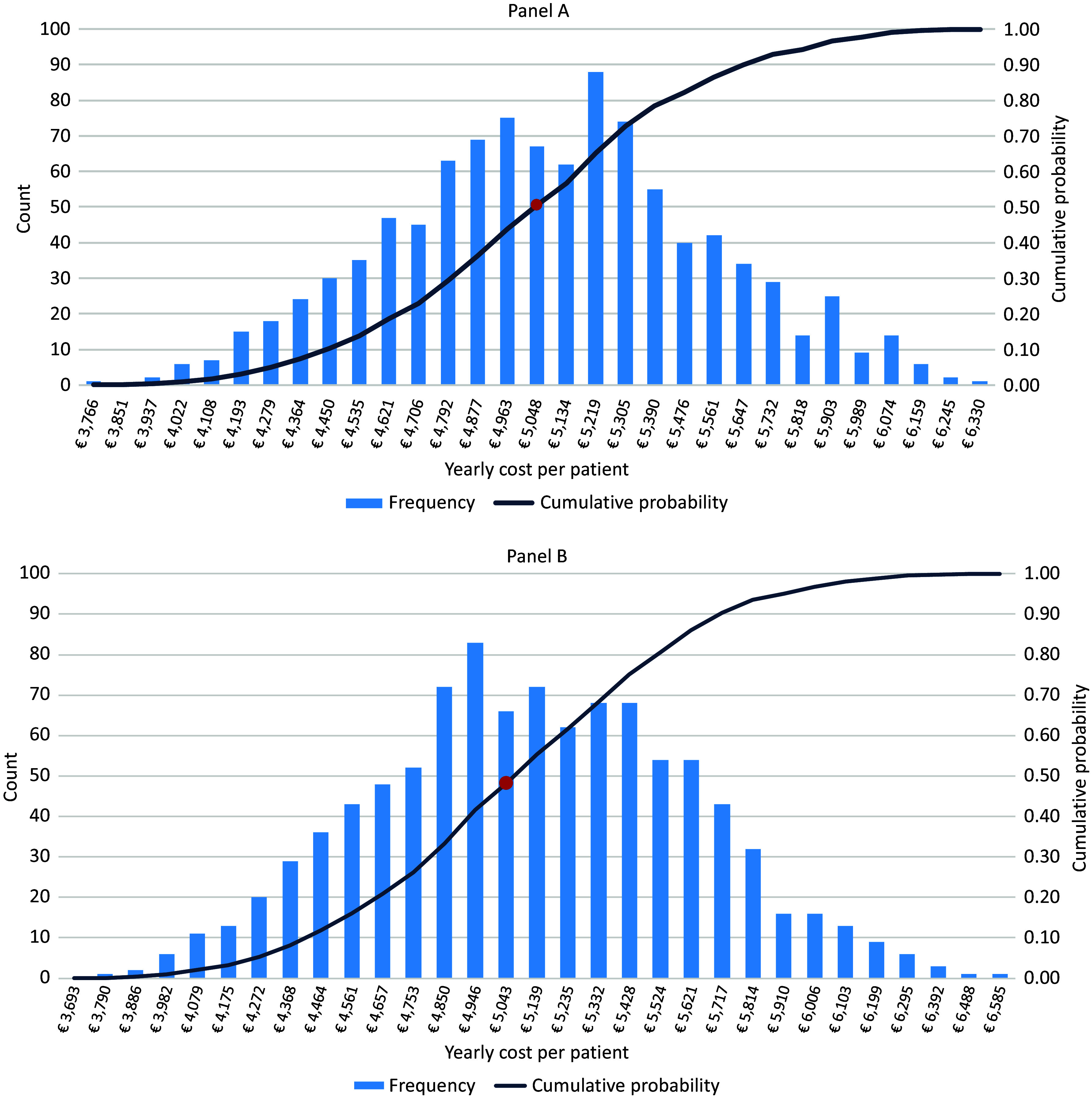
Probabilistic sensitivity analysis – Histogram from Monte-Carlo simulation using triangular distribution (panel A) and beta-PERT distribution.

According to the DSA, the parameters with a substantial impact on cost estimates are the number of PET performed, the probability of hospitalization for major cardiovascular events, the percentage of patients treated with triptorelin, leuprorelin, and degarelix (Figure 2). Using the triangular distribution for parameters, the PSA revealed that there is a 50 percent probability that the yearly cost per patient was lower than or equal to €5,048, and approximately a 23 percent probability that the cost was lower than the result of the base-case scenario (i.e., €4,710) (Figure 3). Similar results were found using the beta-PERT distribution for parameters. In this case, there is a 48 percent probability that the yearly cost per patient was lower than or equal to €5,043, and approximately a 21 percent probability that the cost was lower than the result of the base-case scenario (i.e., €4,710) (Figure 3).

## Discussion

Despite the growing interest in nmCRPC, whose management is rapidly evolving thanks to advancements in treatment with the recent approval of next-generation antiandrogens and improvements in diagnosis with new imaging modalities (e.g., PET) (35;36), little is known about its associated costs. Studies investigating the costs associated with nmCRPC are scant, and are mostly focused on the United States context. The study by Freedland et al. (25) assessed the healthcare costs (namely inpatient, emergency room, outpatient, and pharmacy costs) among patients with nmCRPC in the Veterans Health Administration setting before and after nmCRPC diagnosis. Based on the analysis of electronic health records from January 2007 to August 2017, the authors found that the mean yearly cost per patient (in 2016 US dollars) amounts to $ 15,969 (€ 14,434) before nmCRPC diagnosis and $ 32,935 (€ 29,770) after nmCRPC diagnosis. Wu et al. (26) conducted a retrospective cohort analysis of patients with nmCRPC who progressed to mCRPC, selected from insurance claims data in the United States, and estimated all-cause healthcare costs (including hospitalizations, ER visits, office and outpatient visits, use of skilled nursing facilities, and pharmacy costs). The mean yearly cost per patient (in 2016 US dollars) for nmCRPC patients was estimated between $27,549 (€ 24,902) and $29,192 (€ 26,387). The study by Svensson et al. (27) estimated the healthcare costs associated with advanced PCa in Sweden, using retrospective registry data (2014–2016). Cost items included cancer treatment regimens, hospitalizations, outpatient visits, imaging, and laboratory tests. The authors estimated that the mean yearly cost per nmCRPC patient (in 2018 Swedish krona) is equal to 76,667 SEK, that is, € 7,474. Overall, published studies relied on pre-existing data, retrieved from registries or administrative databases, and focused their analysis on direct healthcare costs, although different cost items were considered.

Our study assessed the direct healthcare costs of nmCRPC in Italy from an NHS perspective through a COI analysis. Owing to the lack of existing evidence on resource consumption for nmCRPC in Italy, we relied on two different methods. First, structured qualitative interviews were carried out with clinical experts (Italian urologists and oncologists) to identify the healthcare resources consumed by nmCRPC patients treated with standard ADT. Then, we performed a structured expert elicitation through an Excel tool to derive unknown resource consumption quantities to inform the COI analysis. Expert elicitation is increasingly used in health economics to support and improve the decision-making process in the absence or scarcity of empirical data (37). Our results suggest that differences in the management of patients are likely to translate also in differences in the consumption of healthcare resources across hospitals. According to the base-case scenario, in which the most likely estimates elicited from experts were used, the yearly cost per nmCRPC patient for the Italian NHS amounts to €4,710, whereas in the most conservative and least conservative scenarios the yearly cost per nmCRPC patient was of € 2,243 and € 8,243, respectively. Based on a DSA, innovative imaging techniques (i.e., labeled choline PET/CT, radioactive tracer prostate-specific membrane antigen PET) frequency and cost weigh in these estimates, followed by frequency of hospitalization for major cardiovascular events. According to the PSA, the estimated cost per year is within €5,048 with 50 percent probability.

Overall, our cost estimates for nmCRPC are lower than those provided in previously published cost studies (25–27). The greatest difference can be noticed with respect to US-based studies, while our results are more in line with those provided in the Swedish study (especially when considering the whole range of estimates). The observed heterogeneity in cost estimates is due to several factors, namely differences in clinical practice, in unit costs (different GDPs and purchasing power), and also in the methods of data collection. Overall, the difference in healthcare resource consumption and costs across different contexts shed further light on the heterogeneity of management of nmCRPC and, therefore, on its economic impact.

Although this study contributes to the evidence on nmCRPC direct healthcare costs for Italy, it has several limitations. First, the opinions drawn from experts may not be fully representative of the Italian clinical practice, as the sample is limited and not randomly selected. However, clinicians were carefully selected based on their recognized expertise in the management of nmCRPC patients and taking into account their geographical distribution, in order to capture heterogeneity in clinical practice in different Italian regions. Moreover, although focused on model-based cost-effectiveness analysis, a recent review of studies performing structured expert elicitation showed that sampling is always purposive and the sample size usually ranges from 2 to 23 experts (37). Second, in the tool we used the three-point estimation method for expert elicitation, which is simpler than other methods (e.g., complementary interval method) although less effective in capturing uncertainty around the expert opinion. However, due to the high number of parameters elicited, we believe that using this method was the only viable option in order not to discourage tool completion and to avoid an excessive burden for clinicians in terms of time and effort. Future work in this area could delve into how patient per-year cost evolves over time, whether it stays stable or rather increases or decreases from the first year to the following. Moreover, we conducted sensitivity analyses, both deterministic and probabilistic, in order to test for heterogeneity in estimates. Third, in the expert elicitation we did not consider new antiandrogens among treatments for nmCRPC (e.g., enzalutamide, apalutamide, and darolutamide). However, at the time of study approval and subsequent tool construction and validation, reimbursement for these molecules in Italy was lacking and the standard of care was represented by ADT. A future update of this work considering actual level of diffusion of prostate-specific membrane antigen ligand positron emission tomography vis-à-vis conventional imaging and new treatments for nmCRPC is needed. Foreseeably, a multicenter observational study would be highly recommended in order to exhaustively capture nmCRPC resource consumption in current clinical practice in Italy. Finally, due to the lack of epidemiological data specifically entailing nmCRPC population for Italy, we were not able to estimate the total economic burden of nmCRPC. A country-based epidemiological study that provides reliable estimates on nmCRPC prevalence and incidence is warranted.

## Conclusion

This study estimated the direct healthcare costs of nmCRPC in Italy by means of qualitative interviews and a structured expert elicitation exercise with clinicians involved in the management of this patients’ population. The results showed that the yearly direct healthcare cost of an nmCRPC patient treated with ADT ranges from €2,243 to €8,243. These findings have the potential to inform decision-making about treatments at the juncture between non-metastatic and metastatic prostate cancer disease.

## Supplementary material

For supplementary material accompanying this paper visit https://doi.org/10.1017/S0266462322003336. click here to view supplementary material
