# Supplementary material for: Direct healthcare costs of non-metastatic castration-resistant prostate cancer in Italy
Source: Int J Technol Assess Health Care. 2023 Jan 6;39(1):e2. doi: 10.1017/S0266462322003336 (PMC11574549; doi:10.1017/S0266462322003336)
Supplement: Supplementary file 1 [file S0266462322003336sup001.zip › S0266462322003336sup001.docx]

Supplementary File 1 Interview guide

*Note. The interview guide provided below is an extract of a larger interview, in which we asked clinicians to provide information on: 1) clinical pathway of nmCRPC patients; 2) resource consumption of nmCRPC patients; 3) health states of nmCRPC patients. In this file we provided only the questions related to resource consumption, being the only relevant section for the present study.*

**Consent**

Before starting the interview, we ask for your consent to use the information collected. We remind you that your participation is voluntary and you can interrupt the interview at any time. Furthermore, we would like to ask your permission to record this interview, in order to ensure the quality of information gathering.

**Introductory remarks**

Thank you for your time […]. The aim of this project is […]. We anticipate that the interview will take no longer than one hour.

Throughout the interview process, we will make written notes on your responses to the various questions. Please bear with us if there are short pauses between questions while we note your replies.

We would like to begin, by learning more about you. What’s your current role, affiliation and experience in the management of prostate cancer?

**Resource consumption**

1. In your experience, what types of healthcare resources are consumed by non-metastatic castration-resistant prostate cancer patients within the current clinical practice? (hint: drugs, laboratory tests, imaging)
2. For each type of resources mentioned, could you please provide additional details? (hint: which drugs? Which laboratory tests? Which imaging?)
3. According to your experience, and within the current clinical practice, which adverse events currently happen? (*Note: the list of adverse events of grade 3/4 experienced by more than 1% of patients from the ADT arm of relevant trials will be presented and discussed with clinicians*)
   1. Fatigue
   2. Rash
   3. Hot flashed
   4. Nausea
   5. Diarrhea
   6. Falls
   7. Constipation
   8. Arthralgia
   9. Asthenia
   10. Loss of appetite
   11. Back pain
   12. Headache
   13. Hematuria
   14. Urinary tract infection
   15. Weight loss
   16. Urinary retention
   17. fracture
   18. Dizziness
   19. hypothyroidism
   20. Hypertension
   21. Major cardiovascular events
   22. Mental disorders
   23. Hepatic insufficiency
   24. Neutropenia
   25. Convulsions
4. How adverse events are currently managed? Which resources are consumed?

**Closing remarks**

We have reached the conclusion of the interview. Do you have any questions or observations that you would wish to make?

If you wish to know about the results of this project we will notify you once the report and related publication will be available.
