# Supplementary material for: Direct healthcare costs of non-metastatic castration-resistant prostate cancer in Italy
Source: Int J Technol Assess Health Care. 2023 Jan 6;39(1):e2. doi: 10.1017/S0266462322003336 (PMC11574549; doi:10.1017/S0266462322003336)
Supplement: Supplementary file 1 [file S0266462322003336sup001.zip › S0266462322003336sup002.docx]

Supplementary File 2 Sample screens of the Excel expert elicitation tool


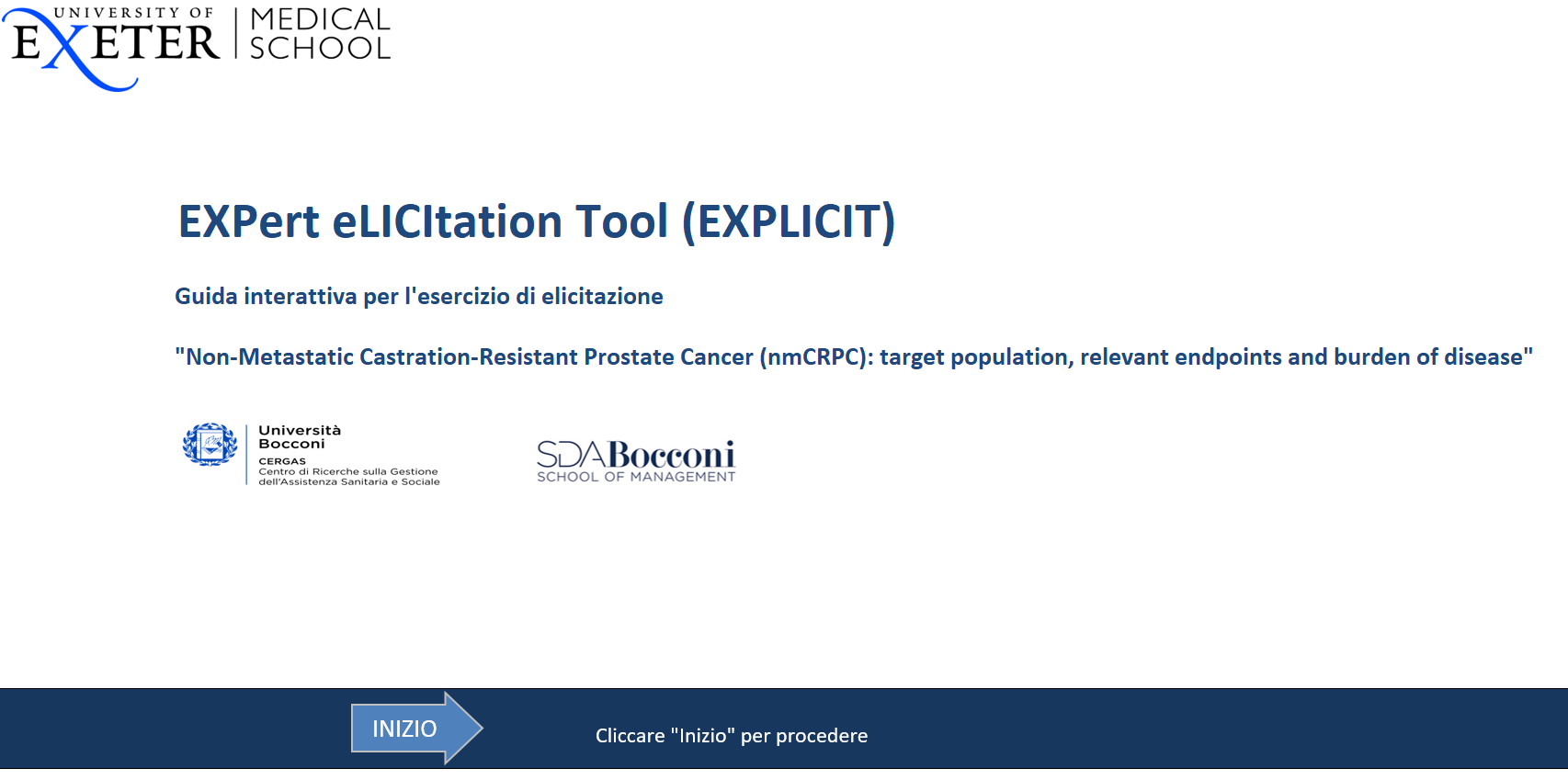


*Note. The figure above is the start screen of the elicitation tool.*


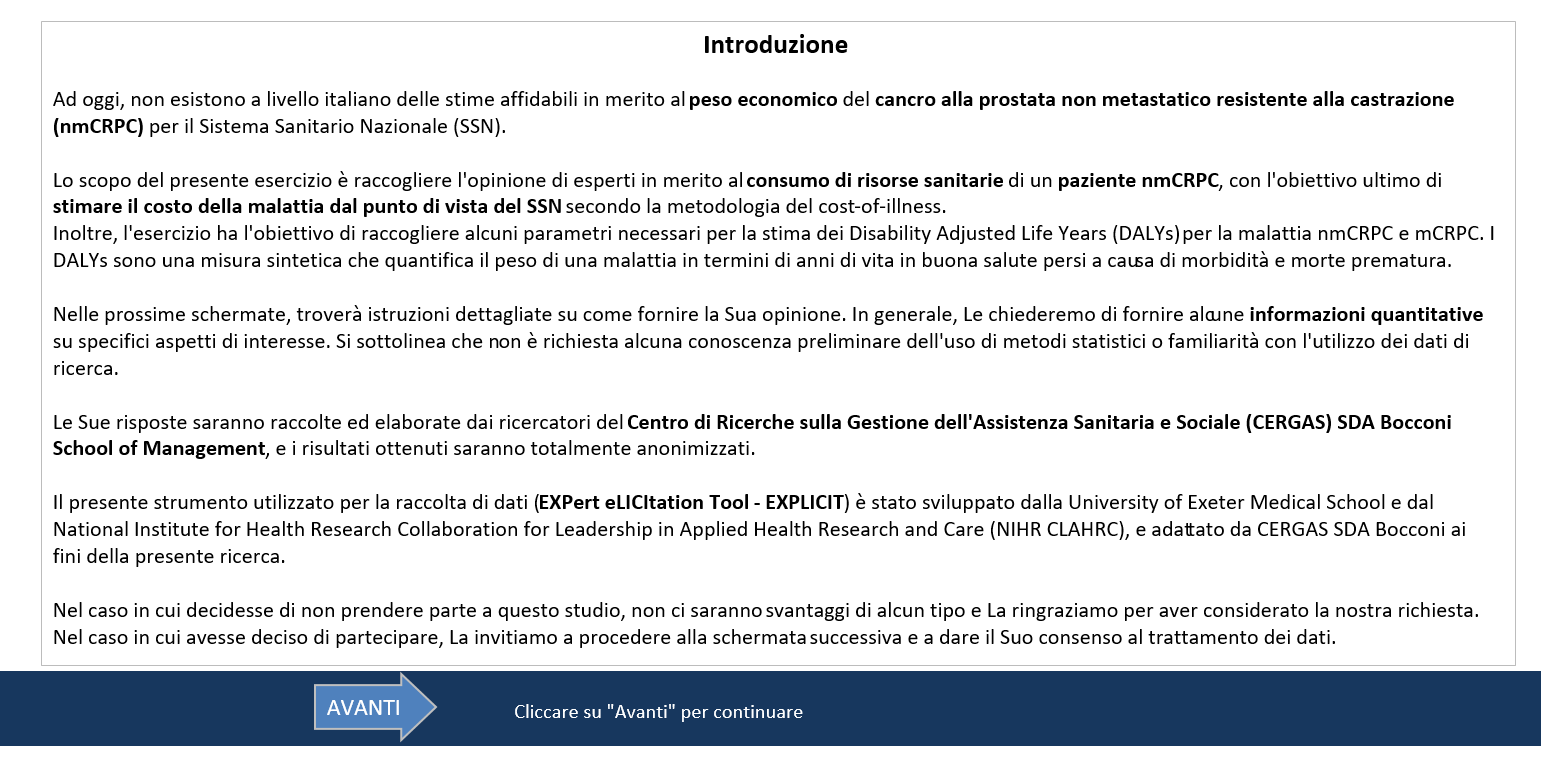


*Note. The figure above is the introduction screen of the elicitation tool, were we provided a short background of the research (rationale and methods) and explained the objectives of the elicitation exercise. Moreover, we underlined that the tool used for data collection (EXPert eLICItation Tool - EXPLICIT) was developed by the University of Exeter Medical School.*


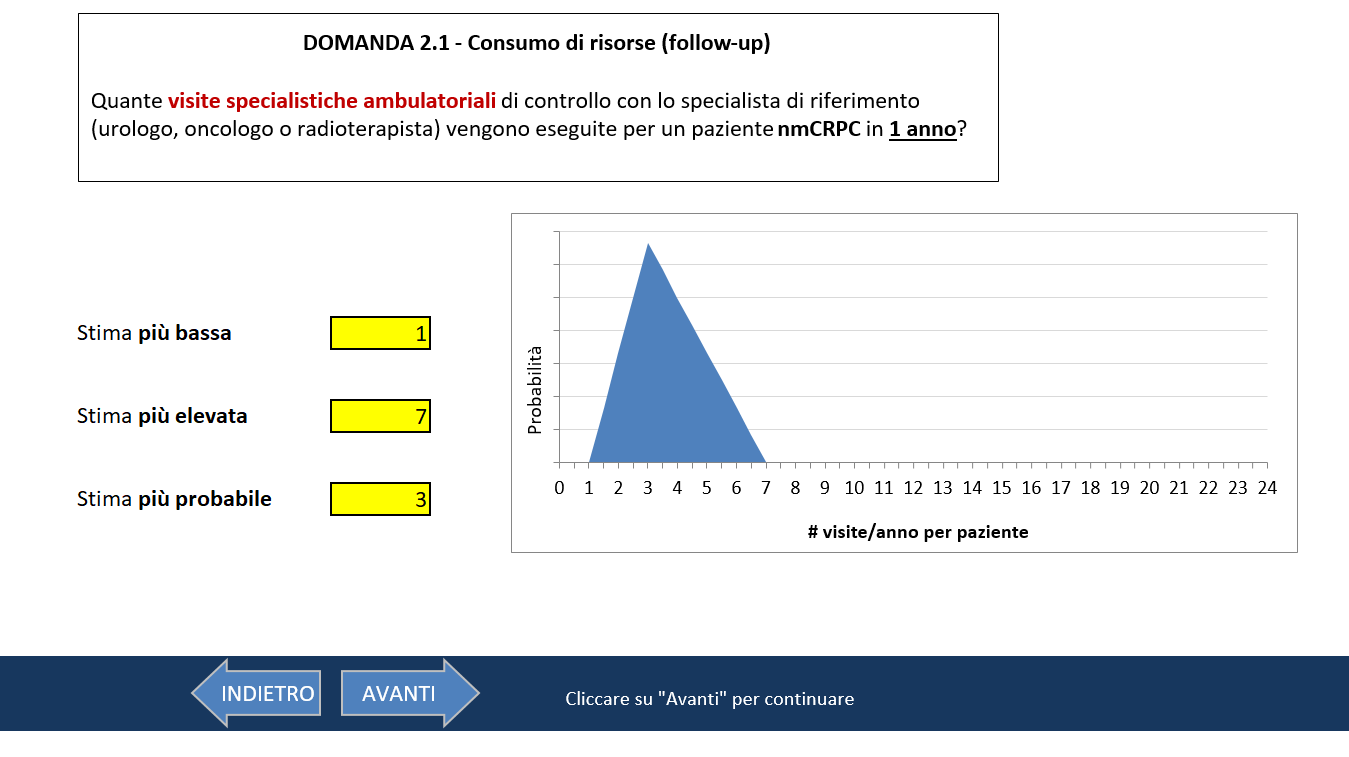


*Note. The figure above is an example of the questions asked to clinicians. Once an expert provided his/her estimates (the lowest, highest and most likely values) about a certain parameter, a graph showing the probability density function of a triangular distribution automatically appeared. The clinician could then revise the estimates provided or proceed to the next section.*
