# Supplementary material for: Direct healthcare costs of non-metastatic castration-resistant prostate cancer in Italy
Source: Int J Technol Assess Health Care. 2023 Jan 6;39(1):e2. doi: 10.1017/S0266462322003336 (PMC11574549; doi:10.1017/S0266462322003336)
Supplement: Supplementary file 1 [file S0266462322003336sup001.zip › S0266462322003336sup003.docx]

Supplementary Table 1 Unit costs of healthcare resources for diagnosis and follow-up of nmCRPC patients

| **Parameter** | **Unit cost** | **Source** |
| --- | --- | --- |
| ***Diagnosis*** |  |  |
| CT scan | € 79.47 | Italian formulary for outpatient services |
| Bone scan | € 113.10 |  |
| PET choline | € 1,071.65 |  |
| PET PSMA | € 1,071.65 |  |
| ***Follow-up (visits and laboratory tests)*** |  |  |
| Outpatient visit | € 20.66 | Italian formulary for outpatient services |
| PSA test | € 7.41 |  |
| Testosterone test | € 9.78 |  |
| Blood count test | € 3.17 |  |
| Blood glucose test | € 1.17 |  |
| Calcium test | € 1.13 |  |
| Vitamin D test | € 15.86 |  |
| Lipid profile test (cholesterol+triglycerides) | € 2.21 |  |
| *Cholesterol* | *€ 1.04* |  |
| *Triglycerides* | *€ 1.17* |  |
| Creatinine test | € 1.13 |  |
| Electrolyte test | € 9.02 |  |
| Transaminases test | € 2.04 |  |
| *GOT* | *€ 1.04* |  |
| *GPT* | *€ 1.00* |  |
| Alkaline phosphatase test | € 1.04 |  |
| Bilirubin test | € 1.13 |  |
