# Supplementary material for: Direct healthcare costs of non-metastatic castration-resistant prostate cancer in Italy
Source: Int J Technol Assess Health Care. 2023 Jan 6;39(1):e2. doi: 10.1017/S0266462322003336 (PMC11574549; doi:10.1017/S0266462322003336)
Supplement: Supplementary file 1 [file S0266462322003336sup001.zip › S0266462322003336sup004.docx]

**Supplementary Table 2 ADT dosage and frequency of administration**

| **Parameter** | **Dosage and frequency of administration** | **Route of administration** |
| --- | --- | --- |
| buserelin | 1.5 mg/day for 7 days | Subcutaneous |
|  | nasal spray (1 bottle/week) from day 8 | Nasal |
| goserelin | 10.8 mg every 3 months | Subcutaneous |
| leuprorelin | 11.25 mg every 3 months | Subcutaneous or intramuscular |
| triptorelin | 11.25 mg every 3 months | Intramuscular |
| degarelix | 80 mg every month | Subcutaneous |
| bicalutamide | 150 mg/day (monotherapy) 50 mg/day (in association) | Oral |
| flutamide | 750 mg/day (3 tablets/day) | Oral |

*Note. As cyproterone acetate was not used by any clinicians, it was excluded from the analysis.*
