# Supplementary material for: Direct healthcare costs of non-metastatic castration-resistant prostate cancer in Italy
Source: Int J Technol Assess Health Care. 2023 Jan 6;39(1):e2. doi: 10.1017/S0266462322003336 (PMC11574549; doi:10.1017/S0266462322003336)
Supplement: Supplementary file 1 [file S0266462322003336sup001.zip › S0266462322003336sup005.docx]

Supplementary Table 3 ADT price per mg and therapy cost per year

| **Parameter** | **Price per mg** | **Therapy cost per year** | **Source** |
| --- | --- | --- | --- |
| buserelin | € 2.13 (subcutaneous) | € 1,286.14 | Farmadati (price per mg), expert opinion |
|  | € 2.48 (spray) |  |  |
| goserelin | € 31.57 | € 1,364 |  |
| leuprorelin | € 24.78 | € 1,115 |  |
| triptorelin | € 26.84 | € 1,208 |  |
| degarelix | € 1.61 | € 1,796 |  |
| bicalutamide | € 0.02 (150 mg) | € 535^*^ |  |
|  | € 0.01 (50 mg) |  |  |
| flutamide | € 0.001 | € 274 |  |

*Note. *W*e assumed 50% in monotherapy and 50% in association
