# Supplementary material for: Direct healthcare costs of non-metastatic castration-resistant prostate cancer in Italy
Source: Int J Technol Assess Health Care. 2023 Jan 6;39(1):e2. doi: 10.1017/S0266462322003336 (PMC11574549; doi:10.1017/S0266462322003336)
Supplement: Supplementary file 1 [file S0266462322003336sup001.zip › S0266462322003336sup006.docx]

**Supplementary Table 4 List of DRGs for the management of serious AEs**

| **Parameter** | **DRG** | **Description** | **DRG tariff (LOS>1 day)** |
| --- | --- | --- | --- |
| Hematuria | 325 | KIDNEY & URINARY TRACT SIGNS & SYMPTOMS AGE >17 W CC | € 1,878 |
|  | 326 | KIDNEY & URINARY TRACT SIGNS & SYMPTOMS AGE >17 W/O CC | € 1,075 |
| Urinary retention | 325 | KIDNEY & URINARY TRACT SIGNS & SYMPTOMS AGE >17 W CC | € 1,878 |
|  | 326 | KIDNEY & URINARY TRACT SIGNS & SYMPTOMS AGE >17 W/O CC | € 1,075 |
| Fractures | 239 | PATHOLOGICAL FRACTURES & MUSCULOSKELETAL & CONN TISS MALIGNANCY | € 3,649 |
| Major cardiovascular events^a^ | | | |
| Acute myocardial infarction | 121 | CIRCULATORY DISORDERS W AMI & MAJOR COMP, DISCHARGED ALIVE | € 4,700 |
|  | 122 | CIRCULATORY DISORDERS W AMI W/O MAJOR COMP, DISCHARGED ALIVE | € 3,377 |
| Hemorrhagic cerebrovascular conditions | 14 | INTRACRANIAL HEMORRHAGE OR CEREBRAL INFARCTION | € 3,891 |
| Ischemic cerebrovascular conditions | 524 | TRANSIENT ISCHEMIA | € 2,543 |
|  | 559 | ACUTE ISCHEMIC STROKE WITH USE OF THROMBOLYTIC AGEN | € 6,073 |
| Heart failure | 127 | HEART FAILURE & SHOCK | € 3,025 |

*Note. ^a^ The list of conditions was retrieved from Hussain et al (2018). In calculating the average DRG tariff, each condition was given equal weight.*
