# Supplementary material for: Direct healthcare costs of non-metastatic castration-resistant prostate cancer in Italy
Source: Int J Technol Assess Health Care. 2023 Jan 6;39(1):e2. doi: 10.1017/S0266462322003336 (PMC11574549; doi:10.1017/S0266462322003336)
Supplement: Supplementary file 1 [file S0266462322003336sup001.zip › S0266462322003336sup007.docx]

Supplementary Table 5 DRG tariffs for the management of serious AEs

| **Parameter** | **DRG tariff** | **Source** |
| --- | --- | --- |
| Hematuria | € 1,477 | Italian DRG tariffs |
| Urinary retention | € 1,477 |  |
| Fractures | € 3,649 |  |
| Major cardiovascular events | € 3,816 |  |
